# Spelling Errors and Shouting Capitalization Lead to Additive Penalties to Trustworthiness of Online Health Information: Randomized Experiment With Laypersons

**DOI:** 10.2196/15171

**Published:** 2020-06-10

**Authors:** Harry J Witchel, Georgina A Thompson, Christopher I Jones, Carina E I Westling, Juan Romero, Alessia Nicotra, Bruno Maag, Hugo D Critchley

**Affiliations:** 1 Department of Neuroscience Brighton and Sussex Medical School Brighton United Kingdom; 2 Department of Primary Care and Public Health Brighton and Sussex Medical School Brighton United Kingdom; 3 Faculty of Media and Communication Bournemouth University Bournemouth United Kingdom; 4 Dalton Maag Ltd London United Kingdom

**Keywords:** communication, health communication, persuasive communication, online social networking, trust, trustworthiness, credibility, typographical errors

## Abstract

**Background:**

The written format and literacy competence of screen-based texts can interfere with the perceived trustworthiness of health information in online forums, independent of the semantic content. Unlike in professional content, the format in unmoderated forums can regularly hint at incivility, perceived as deliberate rudeness or casual disregard toward the reader, for example, through spelling errors and unnecessary emphatic capitalization of whole words (online *shouting*).

**Objective:**

This study aimed to quantify the comparative effects of spelling errors and inappropriate capitalization on ratings of trustworthiness independently of lay insight and to determine whether these changes act synergistically or additively on the ratings.

**Methods:**

In web-based experiments, 301 UK-recruited participants rated 36 randomized short stimulus excerpts (in the format of information from an unmoderated health forum about multiple sclerosis) for trustworthiness using a semantic differential slider. A total of 9 control excerpts were compared with matching error-containing excerpts. Each matching error-containing excerpt included 5 instances of misspelling, or 5 instances of inappropriate capitalization (*shouting*), or a combination of 5 misspelling plus 5 inappropriate capitalization errors. Data were analyzed in a linear mixed effects model.

**Results:**

The mean trustworthiness ratings of the control excerpts ranged from 32.59 to 62.31 (rating scale 0-100). Compared with the control excerpts, excerpts containing only misspellings were rated as being 8.86 points less trustworthy, those containing inappropriate capitalization were rated as 6.41 points less trustworthy, and those containing the combination of misspelling and capitalization were rated as 14.33 points less trustworthy (*P*<.001 for all). Misspelling and inappropriate capitalization show an additive effect.

**Conclusions:**

Distinct indicators of incivility independently and additively penalize the perceived trustworthiness of online text independently of lay insight, eliciting a medium effect size.

## Introduction

### Trustworthiness of Online Health Information: Background, Context, and Importance

As of 2019, 90% of all British adults use the internet at least weekly [1], and as patients, they often search online for health information to solve their medical problems [2]; furthermore, they are likely to be influenced by the online information, including changing their health care decisions and their frequency of ambulatory care visits [3]. In a survey of 2200 adults with chronic health conditions in the United States who were active social media users, 57% used a health condition–specific website (eg, specializing in multiple sclerosis or rheumatoid arthritis) on a monthly basis, and 5% used such sites daily; half of the patients surveyed had asked a health-related question to others online within the previous 6 months, and 87% of those were seeking responses from other patients with the health condition [4].

In response to this explosion of unvetted potential sources providing online health care information that is acted upon, researchers, experts, and medical professionals have repeatedly expressed concern about the inaccuracy of online information and the limited ability of lay consumers to adequately assess its validity [2,5,6]. In particular, it has long been known that when lay users determine whether to use and trust online health care information, they are strongly influenced by nonmedical criteria that experts do not use [7-11]. Broadly, although academics favor a checklist approach of transparency criteria [12], the approach of nonexperts appears to be more variable and situation dependent, and it prioritizes factors such as ease of understanding and the attractiveness of graphic design [9]. More generally, in research on the factors that influence judgments of trust, all aspects of trustworthiness can be relational and depend on the type of person (or group being studied); major relational factors include accessibility, both cognitive as well as physical [13], and correctly accommodating language to the intended audience [14].

Understanding lay assessment of the trustworthiness of online health information is important because false online information presented to the general public, if believed, has the potential to undermine correct medical advice [15], to elicit unhealthy behavior [16], and to influence sociopolitical discourses on health care and other topics [17-19]. Sbaffi and Rowley’s [20] recent review of how laypeople assess the trustworthiness and credibility of online health information concluded that so far, much less research has been performed to understand what interferes with trust (we describe these as penalties to trustworthiness [21]), compared with what causes trust.

Trust and credibility are closely related to one another and information quality, although there remains disagreement among researchers as to the exact definitions of these terms. In general, the definitions emphasize the likelihood of information use, believability, reliability, and dependability [20,22]. Correspondingly, there is little evidence to suggest that the general population reliably make fine conceptual distinctions between trustworthiness and credibility of information.

Online health support often is divided into seeking information or emotional reassurance and can be gender specific (eg, prostate cancer vs breast cancer) and person specific [23,24]. Trustworthiness remains pertinent to online emotional reassurance and sharing, as shown by the many occurrences of large-scale hoaxes designed to manipulate emotions [8]. For example, between 2010 and 2011, a Macmillan cancer forum was inundated with posts in response to an elaborate hoax by a purported mother about her 6-year-old daughter struggling with cancer. On the exposure of the hoax (perpetrated by a lonely 16-year-old girl), many users of the forum—who had formed close online relationships with the supposed mother—refused to believe it had all been a hoax [25]. In light of the range of uses that online health information has for the general public, we contend that it is important to build knowledge of the factors that influence how trust and its absence are formed online beyond source analysis and fact checking. As this study shows, both linguistic and metalinguistic factors affect how trustworthiness is instinctively rated.

### Theoretical Underpinnings for Factors Influencing Trustworthiness

The range of elements that influence online trustworthiness includes issues related to the sources (eg, author identification and the absence of advertising), issues related to the content (eg, a date stamp and inclusion of medical evidence), issues related to design and engagement (eg, inclusion of images), and issues affecting all the above (eg, the absence of typographical errors) [20,26]. For some time, credibility has been subdivided into aspects such as source credibility, message credibility, and media credibility, which strongly influence each other [22]. All such aspects of credibility include accessibility, which can be relational and cognitive as well as physical [13]. More recently, Sun et al [6] have divided the elements that influence consumer evaluation of online health information into 25 *criteria* and 165 *quality indicators*; criteria are rules that reflect notions of value and worth (eg, expertise and objectivity), whereas quality indicators are properties of information objects to which criteria are applied to form judgments (eg, the owner of the website and inclusion of statistics). In line with Diviani et al [5], Sun et al [6] have suggested that indicators can be positive or negative (in terms of trustworthiness), and that consumers’ perceived online health information quality could conceivably be measured by a small set of core dimensions (ie, a few groups of criteria might incorporate many of the quality indicators that explain most of the trustworthiness judgments).

So far, Lederman et al [8] have proposed a five-category model that highlights verification processes via the comparison of different websites or other online statements. This model includes argument quality, source credibility, source literacy competence, and crowd consensus. An extended six-category model [26] proposes that the lay reader may assess some or all of the following: reputation, endorsement, consistency with other sources, self-confirmation (agreement with the reader’s own opinions), persuasive intent, and expectancy violation. For this research, we have adopted Lederman et al’s [8] model of credibility; however, the choice among these models for this research is moot because all the models [5,6,20,22,26] are concordant with the idea that spelling errors will detract from judgments of trustworthiness (ie, they are negative quality indicators).

### Incivility, Literacy Competence, and Errors of Writing Mechanics

Although institutionally produced websites and curated online health content (cf. [6]) will usually be both grammatically correct and civil, the responses by the general public may be uncivil [27], and patient-authored text is known to have many misspellings [28] (and so do discharge summaries written by doctors [29]). Inappropriate capitalization (including inappropriate capitalization of entire words, sometimes called online *shouting*) and misspelling are examples of errors in literacy competence [8] and writing mechanics [30]; writing mechanics is defined as elements of a language that only manifest when communication is in written form. Both inappropriate capitalization and misspelling have been highlighted by qualitative investigations as explicit criteria used by lay readers in judgments of online credibility [8,31]. The rationales given to explain why these two error types undermine trustworthiness are that either (1) the errors imply a lack of intelligence (expertise, ability, authority, and education) [8,32,33] or (2) that they suggest a lack of motivation (objectivity, attention to detail, and conscientiousness) to be trustworthy [34,35]. The term incivility is used to describe this latter lack of motivation or effort to make statements that are compliant with rules of communication. Hargittai [33] distinguishes between spelling errors (errors because of the levels of education or social inequalities) and typographical errors (errors because of accidentally hitting the wrong keys on the keyboard). The fact that these errors are not corrected during proofreading is another form of incivility. Incivility implies a lack of respect for the reader, the platform, and the rules of social exchange [36,37], and it is fundamentally relational. Quantitative research on incivility in the mainstream World Wide Web demonstrates that civil statements are rated as more trustworthy and influential than uncivil ones [38,39].

### Integration Versus Heuristics: Lay Judgments Based on Multiple Cues

The reader who must judge unvetted online health information is faced with a wealth of cues that indicate the degree of trustworthiness, and those cues may have contradictory effects (eg, a cogent message that is misspelled). There are three broad theories for how individuals (both lay and expert) make judgments based on multiple cues. The rational approaches are represented by the information integration model [40], in which an individual accounts for all the different pieces of information by a complex (but often subconscious) mathematical process that is usually based on addition, multiplication, or averaging; extensive observations of integration in judgments occur across cultures and individuals. This is the process that Sun et al [6] allude to when they propose that the consumer integrates the relevant trustworthiness criteria and quality indicators in a “complex cost-benefit analysis.” In 2003, Fogg’s [41] Prominence-Interpretation Theory proposed explicit mathematical relationships for how to predict the effects of multiple factors on credibility, but the theory never detailed how to measure the relevant quantities independently. Computational models typically assume that the elements of incivility (eg, inappropriate capitalization) act together either additively or nonlinearly [42-44], although hypothesis-led proof for this assumption is minimal. The elucidation of this integration is only just starting in the literature [45], and the relative importance of each indicator in this intuitive cost-benefit analysis remains unknown; the relative values for each cue may be elucidated empirically by statistical methods such as regression, but it is unlikely that these values would be explicit or transparent in the minds of most lay decision makers. This is an area that needs to be further researched.

An alternative theory to cost-benefit analysis (ie, for how judgments are made based on multiple cues) is the process-level cognitive perspective [46]; this has been made famous by the heuristics and biases research program from behavioral economics [47]. Heuristics are rapid cognitive strategies or shortcuts (either explicit or subconscious) formulated as practical, bounded rational decision systems for multiple cues that can be more transparent than complex cost-benefit analyses, for example, hierarchical lexicographic decision models [48]. In a take-the-best judgment [49], first, a single cue (the most important one) is searched for in the environment and considered independently of all others, and if a tie or no clear result occurs, then the second most important cue is sought and decided upon, and so on. For example, when deciding whether you have the right of way when driving your car through an intersection, first, you follow the signal of any policemen present, and only if there are no police present, do you seek and consider a traffic light (including a temporary traffic light for construction), and only if there are no traffic lights present, do you then consider static road markings and the positions of the other cars.

In biased heuristics, only a limited subset of information (often only one cue) is used to make a fast and ecological judgment outside of conscious awareness [46]; when biased, these heuristics are used to support preferred or preconceived outcomes. With biased heuristics, the prioritization of the cue, and even the cue’s basic validity for the judgment being made, is dubious. Such biased heuristics have been used to explain seemingly irrational preferences that individuals make in situations involving slot machines and organ donation [50]. Examples of biased heuristic processes include representativeness, where some cues are weighted disproportionately compared with their real representativeness—and availability—where a cue that is easily recalled (such as occurs with recency and news) determines the judgment. None of these judgment models so far proposed have explicitly assessed specific issues within literacy competence.

### Determining the Criteria and Weighting of Judgments Based on Multiple Cues

A key feature of heuristics is that they are typically made subconsciously, and that post hoc explanations for such choices are often self-serving justifications or rationalizations [51]. That is, in the case of heuristics, the decision maker does not have privileged information on how the judgment was made, and furthermore, the decision maker can be wrong about themselves [52]. For example, university students have been shown to greatly overestimate how much they actually learned from excellent lecturers (conflating it with how much they feel they learned, which is discounted by effort and exertion) [53]. This creates potential issues for researchers, in which insight-based techniques (both qualitative interviews and quantitative questionnaires) can lead to judgments and explanations of causes that are inaccurate because of demand characteristics or social desirability [54,55]. It has long been known that when evaluating credibility (eg, website privacy policy), the importance of factors that lay individuals say they would use to make their judgment diverge from the factors they are observed to use [41]. Recent sophisticated measurements suggest that although multiple factors can interact when quantifying perceived credibility (of information for an online health forum), these interactions do not support Petty and Cacioppo’s Elaboration Likelihood Model (ELM) [56]. Furthermore, there is a gap in the literature for any data showing how spelling errors interact with other writing mechanics violations that might also affect trustworthiness [57]. This suggests that lay insight into the influences on their perceptions of trustworthiness are imperfect, and that research on trustworthiness should be supplemented by approaches that do not rely on such insight.

To avoid insight bias from our lay participants, the approach of this study is to compare and contrast *marginal differences* in penalties to trustworthiness elicited by different combinations of literacy or writing mechanics violations. Our experiments on changes in marginal trustworthiness were specifically organized so that the participating healthy volunteers were unaware that the experiment tested the effects of capitalization or misspelling *per se* (with full institutional ethical approval). We did not explicitly ask lay individuals for their beliefs regarding how their process for judging message credibility incorporates quality indicators relating to source credibility and media credibility. That is, instead of asking directly, “How much less would you trust a web post that is misspelled?” we simply presented the participants with some posts that had misspellings and asked the same question as usual, “How trustworthy do you find this information?” while subtly varying misspelling and capitalization (ie, *shouting text*, not acronyms or beginnings of sentences).

### Research Questions

The research questions (RQs) were as follows:

- RQ1: Do errors in writing mechanics and incivility lead to marginal changes (ie, without signposting) in judgments for message trustworthiness?
- RQ2: Are the marginal effects of incivility, such as with inappropriate capitalization, on trustworthiness judgments quantitatively comparable with the known effects of writing mechanics such as spelling errors?
- RQ3: How, if at all, do the effects of writing mechanics (eg, spelling errors) and incivility (eg, inappropriate capitalization) integrate? Is there a ceiling effect or a binary effect in which once a message source is judged as incompetent, no further trustworthiness penalty is added to the judgment (similar to a take-the-best heuristic) [49]? Or is there some additive, multiplicative, synergistic, or otherwise integrative function that increases the penalty on the judgment to a new higher level when both cues are present [40]?

## Methods

### Participants, Recruitment and Ethical Approval

The project was approved by our local ethics committee (Brighton and Sussex Medical School’s Research Governance and Ethics Committee, University of Sussex, approval 16/044/WIT). All experiments were performed according to the Declaration of Helsinki. All individuals provided informed consent via a welcome page in each online study. Participants were recruited via the Prolific website. We specified that the recruitment should focus on UK-based members of the public. Participants consented to participate with the understanding that the research concerned *responses to online text*; none of the advertising, web URLs or experimental information to the participants mentioned that the experiment was related to graphics, formatting, incivility, and spelling. This feature of the advertising was approved by our ethics committee, not least because the paragraphs were not considered misleading or potentially emotionally adverse.

### Study Design Process

The study design was a confirmatory, cross-sectional experiment with a balanced incomplete block design; it was a randomized experiment with lay participants, each of whom experienced only a limited number of the possible options. The total number of experimental excerpts that were tested in the entire cohort was 36; there were 9 excerpts, each in 4 possible versions: no errors, inappropriate capitalization only (Caps only), misspelling only, or the combination of both errors. The stimulus texts (and the versions) are shown in the Multimedia Appendix 1. There were two additional paragraphs that always appeared as the first two stimulus texts, which were training stimuli. The training stimuli were not labeled as being different from other stimuli in any way and were never included in the statistical analyses. The only purpose of the training stimuli was to allow participants to familiarize themselves with the rating task and with the range of the trustworthiness scale: training stimulus 1 was quite believable (mean trustworthiness rating 54.33, SD 23.39; n=301), and training stimulus 2 was less plausible (mean trustworthiness 40.20, SD 25.80; n=301; *P*<.001, paired *t* test).

**Figure 1:**
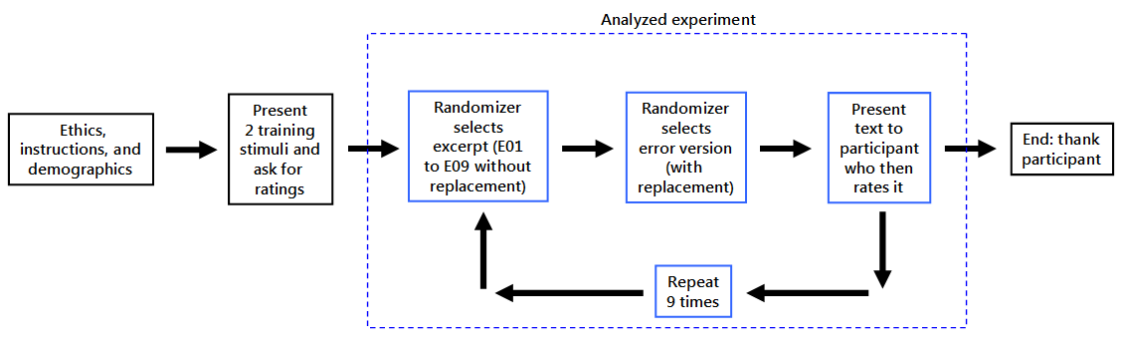
Experiment design: what each participant experiences.

Each participant experienced and rated only 9 (of the possible 36) experimental excerpts, and those 9 included exactly one version of each excerpt, and among those 9, that participant would experience a mix of different error types (Figure 1). For example, participant number 001 experienced the capitalization-only version of excerpt E07 and then the *both errors* (capitalization and misspelling) version of excerpt E02. This double randomization prevented any participant from seeing the same excerpt twice.

The goal was to present to participants a set of coherent text excerpts of similar lengths (70-100 words) on the topic of multiple sclerosis, each excerpt being a coherent answer to a question. The rationale for presenting excerpts in a question-then-answer format was that it was possible to ask whether readers trusted the advice enough to believe it or act upon it. To make these excerpts, a range of comments were found in the public domain (Table 1); these texts often had to be edited substantially to fit within the word count or to avoid explicitly endorsing commercial products (see Multimedia Appendix 2 for a comparison of the original and presented texts). Initially, the excerpts and the presentation system were tested online by a small group of testers, who then provided verbal feedback on the test to the experimental team. After that, a cohort of 40 participants was recruited online to test the paragraphs and demonstrate that the paragraphs elicited similar standard deviations in ratings (19-28 units out of 100) and elicited a wide range of mean trustworthiness ratings (from 20 to 70). These excerpts were to be presented either as they were (*no errors* or the negative control) or in one of the three error versions stated above.

**Table 1:** Sources of excerpts on multiple sclerosis.

Code	Brief topic description	Website	Length
L01	Numerous artificial sweeteners	blogspot.com	78
L02	Hoax about artificial sweeteners	quora.com	81
E01	Triggers of the immune system	healingchronicles.com	81
E02	Programmer’s intelligence	dailystrength.org	89
E03	Epstein Barr virus	medicaldaily.com	92
E04	Avonex patient	quora.com	74
E05	Up there in risk	quora.com	96
E06	Mental exercises	dailystrength.org	85
E07	Vitamin D	quora.com	100
E08	Small risk of Progressive Multifocal Leukoencephalopathy	my-ms.org	90
E09	Half of all people	ms.pitt.edu	71

### Text Interventions

For the error versions of the texts (inappropriate capitalization or misspelling), we wanted to include 5 of the relevant errors per excerpt (a total of 10 errors for the combination of both errors version), with the errors spread throughout the excerpt (rather than bunched together). In excerpts where inappropriate capitalization was required, there would be 5 *sets* of words or phrases. Normally, a set was 1 or 2 words, although one of the 5 sets had to be a 4-word series. The priorities for selecting words to capitalize were (in this order) as follows:

- Adverbs (especially those suggesting extremity such as *very* or *never*)
- Judgments (*rubbish, hopeless,* and *horror*)
- Strong emotions (*worried* and *angry*)
- Words implying danger (*fatal* and *death*)
- Amounts (*all, every*, and *ten*)
- Adjectives (rather than nouns)
- Action verbs (especially gerunds)
- Conjunctions (*and*)

To verify that each word that we capitalized was naturally capitalized on the web, we analyzed words that were capitalized online on Twitter. We used the Claritin corpus, which is a crowdsourced data set of all the Twitter tweets that contained the word *claritin* in the month of October 2012 [58]. This corpus has some 4900 tweets, and we used MATLAB (MathWorks) to find all the words that were in all capitals (which did not have a hashtag or an at-sign in them); this resulted in a list of 343 capitalized words (Multimedia Appendix 3), many of which were short words, acronyms, and internet memes. From this list of words spontaneously capitalized on the web, we selected words in our excerpts to capitalize.

The rationale for how we selected words to misspell was that misspellings should be quite noticeable, and that the meaning of the words should remain clear to the reader even when misspelled. We avoided homonyms and words that looked plausibly English when misspelled. To make sure that misspelled words were noticeable, short words were preferred, or we placed the misspellings in the first syllable of a multisyllable word. In addition, one of the misspelled words had to be in the first 5 words of a paragraph. The misspelled word had to be completely understandable (in the absence of other words or context) even when misspelt. Thus, a misspelled word with missing or added letters should be pronounceable in English (eg, *yu* plainly means *you*). The types of misspellings were as follows:

- Swap one letter for another letter that is next to it on a qwerty keyboard (*pisitive*)
- Double a consonant (*esstimate*)
- Double a vowel or add an extra vowel (*theere*)
- Leave out a vowel (*expsure*)

To verify that each word that we misspelled was naturally misspelled on the web, we searched for the misspelled word along with the words *health* and *forum*; if we could not find at least two examples of a misspelling on online health forums, we did not use it. A complete listing of the misspellings and where we found them online is in Multimedia Appendix 4.

### Study Delivery

The study was presented to participants using the Qualtrics portal, which allows for a wide range of question types and keeps track of answers and total response time. A full description of the survey in the Checklist for Reporting Results of Internet E-Surveys format [59] is included as part of the Multimedia Appendix 5 for this paper. The web-based study welcome page explained in brief what the study was about and what it entailed (estimated 8 min participation time, including reading the ethics and filling in demographics), the ethics of the study (include the ability to withdraw at any time), and a brief complaints procedure. The ethics page explicitly excluded participants aged younger than 18 years or those from vulnerable populations. A pointer to a full-length participant information sheet (3 pages) was shown; the welcome/ethics page had an “I agree” button at the bottom. After the welcome page, participants filled in a brief multiple-choice demographics page, which included questions on gender, age, field of work (eg, health care, agriculture, and retired), and familiarity with the English language/Roman alphabet. All demographic questions included an option for “rather not say.” After the demographics page, participants saw the instructions page and then were launched into the experimental ratings pages.

**Figure 2:**
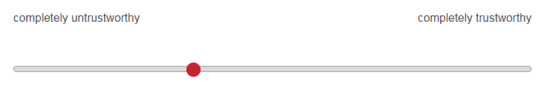
Unnumbered horizontal slider for trustworthiness ratings.

Each ratings page consisted of a short excerpt of text (which was randomized as to whether or not it had the spelling or capitalization errors), followed by a horizontal slider for rating how *trustworthy* the participant found the statement to be; the slider had anchors of *completely untrustworthy* (Figure 2, left) and *completely trustworthy* (Figure 2, right). Although the data collection was numerical (0-100, left to right), there were no numerical cues or tic marks seen by the participants. The instructions to the participants for the trustworthiness ratings were, “If you find something trustworthy, you would be prepared to act upon it; an untrustworthy statement you would ignore, and a rating in the middle represents information where you would want more proof or confirmation that it is correct.” As explained in the instructions to participants, each stimulus excerpt was written as if it was an answer to a question written on an unmoderated health forum, with a specific focus on multiple sclerosis. Multiple sclerosis was chosen as a topic because the information was obviously important, but healthy participants would be unfamiliar with the veracity of each statement; thus, we predicted that the trustworthiness ratings would be more susceptible to nonverbal cues. The questions were as follows: (1) Is multiple sclerosis preventable? (2) How risky is Tecfidera as a treatment for multiple sclerosis? and (3) Does multiple sclerosis decrease intelligence/IQ? The nominal responses to these questions were the experimental stimulus excerpts being rated.

### Study Design, Analysis, and Statistics

#### Sample Size

To detect a difference in rating scores between two groups (ie, no errors vs misspellings, inappropriate capitalization, or both), with 80% power at the 5% significance level, assuming the standard deviation within each group is 25 and the difference between groups is 15 (equivalent to a medium effect size of 0.517), would require 60 participants per group (120 total). Each participant was asked to rate 9 text excerpts, randomly divided between the 4 conditions, and assuming an intraclass (ie, within participant) correlation coefficient of 0.185 gives a design effect of 2.48. The product of the design effect and the sample size for a nonrepeating experiment is 2.48×120≈300 total.

#### Modeling

Linear mixed effects (LME) models were fitted in Stata version 16.0 using the *mixed* command. Residuals were checked for normality and homoscedasticity at the cluster and individual levels. Where the assumption of residual homoscedasticity was not appropriate, robust standard errors were used to allow for the calculation of appropriate 95% CIs and *P* values [60].

## Results

### Differences Between the Paragraphs

**Figure 3:**
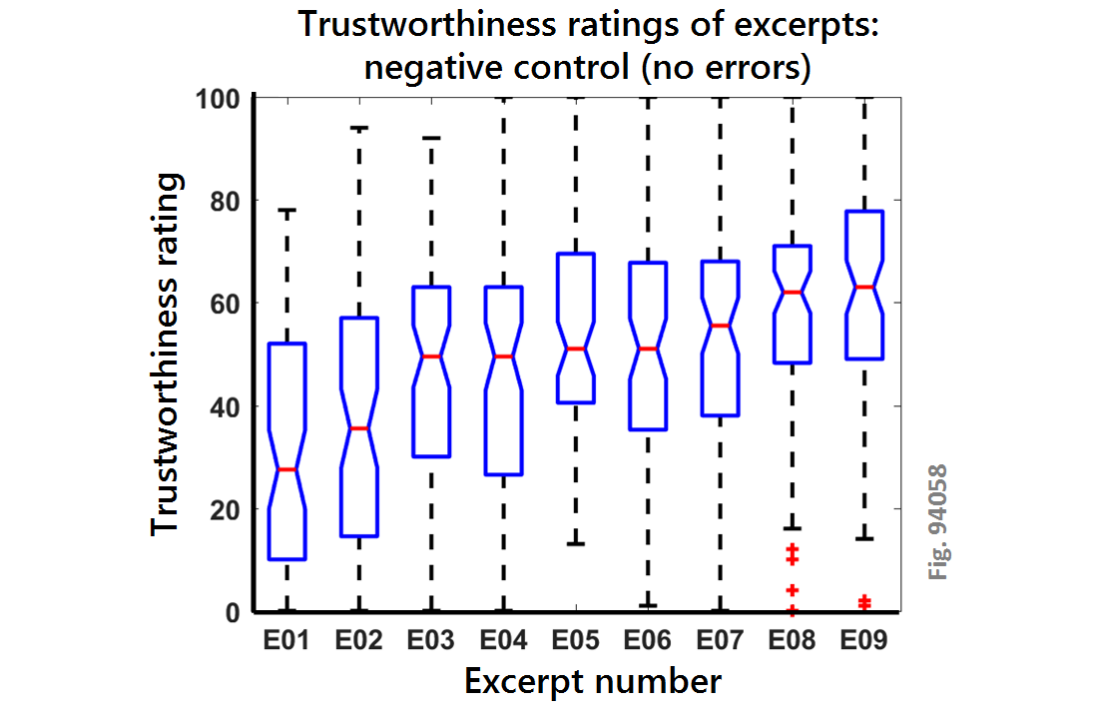
Trustworthiness ratings of the different excerpts in the negative control (no errors) condition. For each box, N=75.

We ran an experiment in which we gathered data from 301 volunteers, each making 9 experimental observations (2709 ratings in total). We estimated that this task (rating 11 excerpts plus instructions and demographics) would take 8 min; in fact, it took a mean time of 6.25 min (mean 375.74 seconds, SE of mean 12.94 seconds). The median trustworthiness ratings of each of the excerpts (E01 to E09) in the negative control condition (ie, without any errors or incivility) are shown in the box and whisker plot in Figure 3. On each box, the red line in the center indicates the median, and the bottom and top edges of the box indicate the first and third quartiles, respectively. When the notches for two boxes do not overlap, this implies that the true medians do differ with 95% CI. The whiskers extend to the most extreme data points not considered outliers, whereas outliers are shown using red plus signs; outliers are any points that fall more than 1.5 times the IQR away from the main box. Excerpt 01 generally elicits low ratings of trustworthiness (median 27.5), whereas excerpts 08 and 09 generally elicit high ratings of trustworthiness (medians 62 and 63, respectively). As illustrated by the nonoverlapping notches, these two sets of excerpts elicit significantly different ratings of trustworthiness, which has been true in all our previous cohorts rating these excerpts for trustworthiness (data not shown). Excerpts 03, 04, 05, and 06 all elicited median ratings in the middle range from 45 to 55, whereas excerpt 02 is a transitional excerpt between E01 and the middle range and E07 is a transitional excerpt between the middle range and the most trustworthy excerpts.

### Cumulative Distributions Shifted Left by Errors

**Figure 4:**
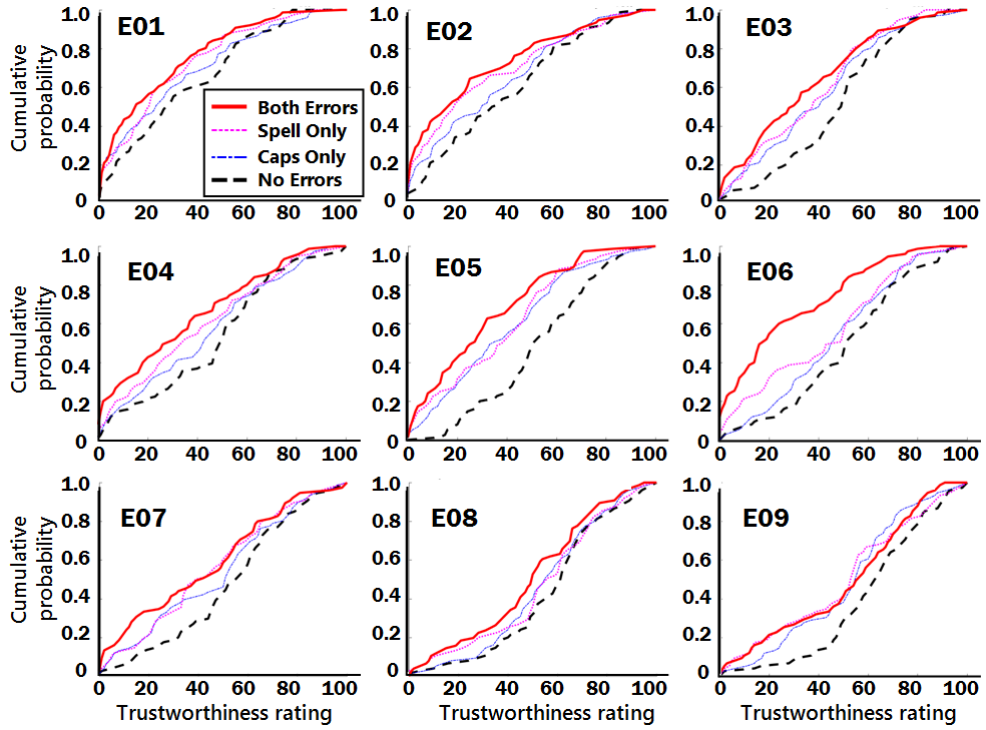
Cumulative probability distribution plots for each excerpt (E01 to E09), comparing the alternative writing errors (lighter colored lines) with negative control (thick, black dashed line).

Figure 4 shows how the errors in writing mechanics and incivility led to changes in the cumulative probability distributions for each of the excerpts. As expected, for each excerpt, compared with the negative control with no errors (black, thick dashed line), all three alterations (inappropriate capitalization (blue, thin dot-dash line), misspelling (magenta, thin dotted line), and both errors together (red, thick solid line) led to a decrease in the ratings of trustworthiness (ie, a leftward-upward shift in the curve). For most of the excerpts, at most points on the cumulative distribution curve, the combination of errors (both inappropriate capitalization and misspelling) led to decreases in trustworthiness ratings (ie, a shift left and up) compared with either of the single errors. That is, the lines for inappropriate capitalization only (blue, thin dot-dash) and misspelling only (magenta, thin dotted) fall between the thick black dashed line (no errors) and the red solid line (both errors); this result suggests that the two errors together have an additive or integrative effect.

### Mixed Linear Effects Model With Four Conditions of Alteration

We tested these data in a mixed linear effects model (model 1), where trustworthiness rating was the dependent variable, whereas alteration (no errors/misspellings/inappropriate capitalization/both misspellings and inappropriate capitalization) and excerpts (1-9) were included as fixed effects. A random effect for participant was included in the model to account for clustering of observations within volunteers. The reference group/condition for this model was E05, with no literacy errors. This model (and the following model) was calculated with robust standard errors [60] to allow for the heteroskedasticity of the residuals. The results of model 1 are shown in Table 2. The intracluster correlation (correlation within the individuals) coefficient estimate for model 1 is 0.334 (95% CI 0.287 to 0.384).

There is strong evidence against each of the null hypotheses that inappropriate capitalization only (result 1), misspelled only (result 2), and both errors (result 3) do not affect trustworthiness ratings compared with the negative control group; stated positively, our data suggest that there is a statistically significant penalty to trustworthiness for inappropriate capitalization (result 1), misspelling (result 2), and for both errors together (result 3). Inappropriate capitalization reduces trustworthiness ratings by −6.41 (95% CI −8.96 to −3.86), and misspelling reduces trustworthiness ratings by −8.86 (95% CI −11.61 to −6.11). The effect on trustworthiness ratings of combining both inappropriate capitalization and misspellings together is −14.33 (95% CI −17.11 to −11.55), which appears to be an additive effect.

Our further analysis aimed to test whether there was likely to be either an additive or integrative effect [40] of combining inappropriate capitalization and misspelling. Such an effect should lead to a significantly larger trustworthiness penalty when both error types are combined compared with either error individually. To test for this, an alternative specification of the LME model of the same data was formulated (model 2). In model 2, the condition *both errors* was specified as an interaction between 2 binary variables, (1) misspelling and (2) capitalization errors, instead of considering all the errors as 4 conditions in a categorical independent variable. The specification of the rest of the model was identical to model 1. In this model (Table 3), the main effects and an interaction between them provide no evidence for an interaction effect between the 2 variables; that is, the main effects for inappropriate capitalization only and for misspelled only were as in the original model 1 (Table 2), whereas the coefficient for the interaction was not significantly different from zero. This supports the interpretation that the effects of the two error types are additive, rather than partially summative or synergistic.

**Table 2:** Mixed effect model 1 for trustworthiness rating, with errors and excerpts as fixed effects and a random effect for the clustering of data by participant.

Categorical variables		Coefficient	Robust SE	*P* value	95% CI
**Alteration**					
	Caps only	−6.41	1.30	<.001	−8.96 to −3.86
	Misspelled	−8.86	1.40	<.001	−11.61 to −6.11
	Both errors	−14.33	1.42	<.001	−17.11 to −11.55
**Excerpt**					
	E01	−11.94	1.71	<.001	−15.28 to −8.59
	E02	−8.29	1.58	<.001	−11.38 to −5.19
	E03	−0.70	1.56	.65	−3.77 to 2.36
	E04	0.03	1.54	.98	−2.99 to 3.05
	E06	1.80	1.62	.27	−1.39 to 4.98
	E07	6.19	1.71	<.001	2.83 to 9.55
	E08	14.75	1.53	<.001	11.75 to 17.75
	E09	14.22	1.46	<.001	11.36 to 17.09
Constant		47.06	1.53	<.001	44.06 to 50.06

**Table 3:** Model 2: Alternatively specified linear mixed effect model with 2 binary variables for capitalization and spelling errors and an interaction term to account for combining both types of error (all unlisted values are identical to above).

Categorical variables		Coefficient	Robust SE	*P* value	95% CI
**Alteration**					
	Caps only	−6.41	1.30	<.001	−8.96 to −3.86
	Misspelled	−8.86	1.40	<.001	−11.61 to −6.11
Interaction		0.94	1.68	.58	−2.34 to 4.24

The output for model 1 shows the following comparisons: (1) capitalization only versus no errors, (2) misspelled only versus no errors, and (3) both errors versus no errors. Table 4 shows the additional comparisons between (4) capitalization only versus misspelled only, (5) both errors versus misspelled only, and (6) capitalization only versus both errors.

Comparison 4 indicates there is weak evidence (*P*=.06) against the null hypothesis of no difference between the effects of capitalization only and misspelled only. That is, misspelled only leads to a larger trustworthiness penalty by −2.45 (95% CI −5.02 to 0.12) compared with capitalization only. There is strong evidence against the null hypothesis of there being no difference between the effects of capitalization only versus both errors combined (comparison 6). Compared with capitalization only, the combination of both errors significantly reduces trustworthiness ratings by a further −7.92 (95% CI −10.28 to −5.56). Similarly, there is strong evidence against the null hypothesis of there being no difference between the effects of misspelled only and the combination of both errors (comparison 5). Compared with misspelling only, the combination of both errors leads to a further penalty to trustworthiness ratings of −5.47 (95% CI: −7.83 to −3.11).

**Table 4:** All possible alterations tested in between-group comparisons (model 1).

Comparison	Coefficient	Robust SE	*P* value	95% CI
(1) Caps only versus no errors	−6.41	1.30	<.001	−8.96 to −3.86
(2) Misspelled versus no errors	−8.86	1.40	<.001	−11.61 to −6.11
(3) Both errors versus no errors	−14.33	1.42	<.001	−17.11 to −11.55
(4) Caps only versus misspelled	2.45	1.31	.06	−0.12 to 5.02
(5) Both errors versus misspelled	−5.47	1.20	<.001	−7.83 to −3.11
(6) Caps only versus both errors	7.92	1.20	<.001	5.56 to 10.28

### Statistically Significant Differences Between the Excerpts

In model 1, when compared with E05, the effect of the various excerpts’ contents on trustworthiness ratings varies from −11.93 to 14.75 (a range of 26.68). This range is roughly twice as large as the effect of both errors (−14.33), suggesting that the errors in incivility and writing mechanics that we tested can together have an overall effect of nearly half of the effects of the content of the excerpts we tested.

## Discussion

### Original Contributions

This study sought to quantitatively determine how two different errors of writing mechanics (contributing to incivility) combine to penalize subjective ratings of trustworthiness in the medically relevant context of materials typical of an unmoderated online health forum. Using an LME model of a suitably powered study, we found that all three interventions (inappropriate capitalization, misspelling, and the combination of the two) were significantly different from the negative control (no added errors or incivility), which clearly answers RQ1. The data also show that (for these 70- to 100-word long excerpts about multiple sclerosis), the trustworthiness penalty for 5 instances of inappropriate capitalization was of a similar magnitude to the penalty for 5 instances of misspelling. Note that there was a trend for the penalty of misspellings to be larger, but as a generalized rule, the 2 are similar in magnitude, and the precise difference will depend on exactly how many words and which words are capitalized or misspelled. This finding answers RQ2. Finally, with a combination of different LME models, the data show that the combination of two different types of errors had a significantly greater trustworthiness penalty than either of the error types alone, and that the effect in this study was almost perfectly additive (RQ3); thus, the effects of the combination of errors were integrative [40] rather than a simplified heuristic such as take-the-best [48], multiplicative, or affected by ceiling effects in this study. This begins to answer the question recently posed of how spelling interacts with other quality indicators on credibility [57]. To the best of our knowledge, this is the first study that was specifically designed to test and quantify these kinds of specific additive effects on trustworthiness independently of the lay participants’ insight.

We also showed that the stimulus excerpts that we designed are appropriate for studies that test the trustworthiness of online health information independently of lay insight. Although our study did not preclude lay insight (ie, participants might notice that some words were misspelled), the study was not dependent on such insight, which is useful for interrogating intuitive evaluations of information (ie, cost-benefit analyses [6]). In this study, the semantic content of the excerpts engendered consistent effects on trustworthiness ratings (at least among this type of online psychology experiment cohort, see Figure 3). In addition, this is the first time that numerical values have been gathered for the isolated effects of inappropriate capitalization.

### The Results in Context

This study reaffirms an earlier observation that incivility decreases message credibility [38,39]. As suggested previously, inappropriate capitalization (shouting) is histrionic and induces a strong effect of incivility on readers’ subjective ratings [37]. In our study, the effect of shouting text showed a trend for eliciting a smaller trustworthiness penalty than the effect of similar quantity of misspelling. One could easily speculate about new experiments where we might change the quantities of literacy errors; our experiment used either 5 misspellings or 5 shouting phrases, but one could run experiments to titrate errors, for example, to determine the relative effects of 3 misspellings or 10 inappropriate capitalizations. Furthermore, the effects of text shouting may be moderated by whether the statements are controversial [38]. We deliberately chose statements about multiple sclerosis that would be unfamiliar to the general public. This lack of familiarity makes the content and context not particularly emotional or controversial, likely weakening the effect of inappropriate capitalization.

The debate between how accessibility (ie, cost and speed) versus information quality (ie, accuracy and presumed benefit) quantitatively affect the use of (and search for) information continues [13]. Categorical frameworks have long been proposed to provide a theoretical underpinning for the factors, grouping specific elements that influence perceptions of trustworthiness in a variety of ways. The most well-known 2-category grouping of factors affecting how people respond to communication is Petty and Cacioppo’s ELM for persuasion [56], in which elements contributing to a central pathway (eg, argument quality) are complemented by seemingly less rational elements that contribute to a peripheral pathway (eg, website design) [20,41]. Another two-category persuasion model that has been used to explain online trustworthiness is Chaiken’s dichotomy of heuristics versus systematic information [61,62]. In both the Heuristic-Systematic Model and the ELM, diminishing motivation/involvement (or diminishing user dependency [45]) is associated with a switch from focusing on the effortful systematic evaluation of information (quality) to low-effort heuristics (accessibility).

In purchasing decisions, the type of product affects how strongly grammar and mechanics errors affect credibility [30]. In particular, *experience goods* (nontechnical items such as body lotion that are used personally) are more affected by grammar and writing mechanics than *search goods* (technical items such as printers). The implication is that when objective signals about trustworthiness are absent, heuristics play a stronger role [56,61]. In this study, where laypersons made judgments about unfamiliar medical issues, we might expect to find a stronger response to misspelling and capitalization. A necessary future approach is to repeat this experiment with multiple sclerosis patients who would exhibit user dependence when evaluating the statements [45].

### Limitations

Our study had several important constraints. We deliberately tested laypersons’ judgments on unfamiliar ideas about multiple sclerosis and showed that literacy errors can have a strong effect. Nevertheless, this effect may be smaller in a cohort that is more dependent on knowing the information. In particular, if readers are dependent, then preexisting points of view and feeling of *homophily* [63] will influence perceived credibility, in a way that would not influence the general public with less interest in statements for and about multiple sclerosis.

It is also notable that in this study, the highest mean trustworthiness rating was 62.3 (0-100 scale), despite the statement being medically correct. Multiple factors may account for why this mean rating is not higher for trustworthiness. For example, participants in this psychology experiment saw the statements *in vacuo*, so that they could not verify the statement, check source credibility, or look for crowd consensus [8]. In real-life situations, other aspects of trustworthiness may dominate, and among some individuals, there may be ceiling and floor effects within this dataset, given the very wide standard deviations.

### Conclusions

Incivility and literacy competence are proposed factors in how lay web users assess the trustworthiness of online health information. These results support Lederman et al’s [8] theory of credibility assessment in online forums; the results also fit with Anderson’s [40] description of integrative assessment of multiple cues. Here, we have shown that literacy competence errors have additive effects. The additive effects are strikingly precise. This implies that when people make seemingly rapid judgments about the trustworthiness of online text—no more than rough estimates—that their intuitive estimates seemingly add up quite accurately, at least at the population level. This completely vitiates binary models of judgment where a writer is judged as either competent or incompetent, with no further penalty for additional errors in writing mechanics [57]. The implication for writers is that for this level of errors (5 in 1 paragraph), multiple errors can add up.

Many other factors also contribute to trustworthiness, notably the argument quality of the content (logic), verification with other sources, reference credibility, and crowd consensus [8]. How these additional factors interplay with literacy competence will require further extensive research. A start would be to determine how persons with multiple sclerosis (with similar education levels to our current cohort) respond to these stimulus excerpts, as those readers would understand the information in a more self-relevant context.

## Appendix

Multimedia Appendix 1 Stimulus Paragraphs and errors list.

Multimedia Appendix 2 Excerpts with original sources.

Multimedia Appendix 3 claritinCapitalizedWords.

Multimedia Appendix 4 Misspellings and Verifications.

Multimedia Appendix 5 Supplementary Methods: CHERRIES.

## Abbreviations

ELM: Elaboration Likelihood Model
LME: linear mixed effects
RQ: research question
