# Correction: Spelling Errors and Shouting Capitalization Lead to Additive Penalties to Trustworthiness of Online Health Information: Randomized Experiment With Laypersons

**DOI:** 10.2196/29452

**Published:** 2021-04-13

**Authors:** Harry J Witchel, Georgina A Thompson, Christopher I Jones, Carina E I Westling, Juan Romero, Alessia Nicotra, Bruno Maag, Hugo D Critchley

**Affiliations:** 1 Department of Neuroscience Brighton and Sussex Medical School Brighton United Kingdom; 2 Department of Primary Care and Public Health Brighton and Sussex Medical School Brighton United Kingdom; 3 Faculty of Media and Communication Bournemouth University Bournemouth United Kingdom; 4 Dalton Maag Ltd London United Kingdom

In “Spelling Errors and Shouting Capitalization Lead to Additive Penalties to Trustworthiness of Online Health Information: Randomized Experiment With Laypersons” (J Med Internet Res 2020;22(6):e15171) one error was noted after publication.

One reviewer of this paper, L Sbaffi, was listed twice in the originally published paper due to a system error. The duplicate instance of the reviewer’s name has been removed from the corrected version.

The correction will appear in the online version of the paper on the JMIR Publications website on April 13, 2021, together with the publication of this correction notice. Because this was made after submission to PubMed, PubMed Central, and other full-text repositories, the corrected article has also been resubmitted to those repositories.

