# Supplementary material for: Spelling Errors and Shouting Capitalization Lead to Additive Penalties to Trustworthiness of Online Health Information: Randomized Experiment With Laypersons
Source: J Med Internet Res. 2020 Jun 10;22(6):e15171. doi: 10.2196/15171 (PMC7315370; doi:10.2196/15171)
Supplement: Multimedia Appendix 2 [file jmir_v22i6e15171_app2.doc]

MS excerpts used in our questionnaires with their sources

For each excerpt, the text as it appears in the experiment is shown, followed by the original online source; those parts of the online source that are highlighted in yellow are either identical to, or nearly the same, as some of the text that we used in the experimental excerpt.

**Practice Paragraph 1: "Numerous"**

**Are the artificial sweeteners in diet soda bad for people with multiple sclerosis?**

Experimental Excerpt:

Numerous studies have shown that consuming aspartame can significantly elevate excitotoxins in the blood. When aspartate (as aspartame) is combined in the diet with monosodium glutamate (MSG), blood levels of endotoxins become several fold higher than normal. With the Blood Brain Barrier damaged, as in MS, these excitotoxins can freely enter the site of pre-existing damage, greatly magnifying the damage. A diet high in excitotoxins, such as aspartame, can convert benign, subclinical neural damage into full-blown clinical MS.

78 words, 148 syllables, 453 characters w/o spaces reading time: 23 seconds

Original:

http://sweetremedyfilm.blogspot.com/p/connection-between-ms-and-aspartame.html

The Connection Between MS and Aspartame

Recently, much controversy has surrounded a claim that aspartame may produce an MS-like syndrome. A current review of recent peer-reviewed scientific studies have disclosed a pathophysiological mechanism to explain this connection. As far back as 1996 it was shown that the lesions produced in the myelin sheath of axons in cases of multiple sclerosis were related to excitatory receptors on the primary cells involved called oligodendroglia. Recent studies have now confirmed what was suspected back then. The loss of myelin sheath on the nerve fibers characteristic of the disease are due to the death of these oligodendroglial cells at the site of the lesions (called plaques). Further, these studies have shown that the death of these important cells is as a result of excessive exposure to excitotoxins at the site of the lesions.

Normally, most of these excitotoxins are secreted from microglial immune cells in the central nervous system. This not only destroys these myelin-producing cells it also breaks down the blood-brain barrier (BBB), allowing excitotoxins in the blood stream to enter the site of damage. Aspartame contains the excitotoxin aspartate as 40% of its molecular structure. Numerous studies have shown that consuming aspartame can significantly elevate the excitotoxin level in the blood. There is a common situation during which the excitotoxin exposure is even greater. When aspartate (as aspartame) is combined in the diet with monosodium glutamate (MSG) blood levels are several fold higher than normal. With the BBB damaged, as in MS, these excitotoxins can freely enter the site of injury,greatly magnifying the damage. So, we see that dietary excitotoxins, such as aspartame and MSG, can greatly magnify the damage produced in multiple sclerosis. Likewise, excitotoxins have been shown to breakdown the BBB as well.

Of equal concern is observation that we know that about 10% of the population (based on autopsy studies of elderly) have MS lesions without ever developing the full blown disease, a condition called benign MS. A diet high in excitotoxins, such as aspartame, can convert this benign, subclinical condition into full-blown clinical MS. The amount of excitotoxins consumed in the average American diet is considerable, as shown by several studies. In addition, the toxin methanol is also in the aspartame molecule. Methanol is a axon poison. Combined toxicity of the aspartate and the methanol adds up to considerable brain toxicity and can convert benign, subclinical MS into full-blown MS. Once the MS becomes full-blown, further consumption of excitotoxins magnifies the toxicity, increasing disability and death.

Recent studies have also shown that even single exposures to these food-based excitotoxins can produce prolonged worsening of neurological lesions. In addition, it has been demonstrated that autoimmune reactions (as occurs with MS) greatly magnifies the toxicity of aspartate and glutamate (the excitotoxins). We also know liquid forms of excitotoxins are significantly more toxic because of rapid absorption and higher blood levels. In the face of this connection between excitotoxicity and the pathophysiology of MS, it would be ludicrous to allow further use of this excitotoxin containing sweetener..

=============================================

**Test Paragraph 2: "Hoax"**

**Are the artificial sweeteners in diet soda bad for people with multiple sclerosis?**

Experimental Excerpt:

There is an online hoax that makes this allegation.   Many people make many claims about aspartame. I like to say that the number one side effect of aspartame is testing in laboratory rats. But aspartame and other sweeteners do not pose any sort of health risk. It is a common scapegoat of fear-mongers.  There is a small chance that artificial sweeteners can have an effect on metabolism that hinders weight loss (and the effect would be minuscule compared to actual sugar).

81words, 130 syllables, 392 characters w/o spaces reading time: 25 seconds

Original:

https://www.quora.com/Is-diet-soda-bad-for-people-with-multiple-sclerosis-Ive-heard-that-the-artificial-sweeteners-can-contribute-to-the-damage-caused-by-the-disease

There is an online hoax that makes this allegation.

Many people make many claims about aspartame. I like to say that the number one side effect of aspartame is testing in laboratory rats. But aspartame and other sweeteners do not pose any sort of health risk. It is a common scapegoat of fear-mongers.

There is some chance (a small chance of it existing at all and if it does the effect would then vary by individual case) that artificial sweeteners can have an effect on metabolism that hinders weight loss (and the effect would be minuscule compared to actual sugar).

==========================================

**Excerpt 01: "Trigger"**

**Is multiple sclerosis preventable?**

Experimental Excerpt:

Yes, MS is Completely Preventable.  To a large degree, ms is a trigger and response problem. Something triggers the immune system and the immune system responds by attacking the nervous system.  Preventing ms involves being careful to actually reduce exposure to these triggers by, for instance, eating organically grown high quality food, refraining from smoking, using less toxic cleaning products, and basically living a more natural, simpler lifestyle that does not involve constantly bombarding one’s body with reasons to attack itself.

81 words, 143 syllables, 459 characters w/o spaces reading time: 25 seconds

Original:

https://healingchronicles.com/2013/03/20/yes-ms-is-completely-preventable-heres-how/

Yes, MS is Completely Preventable. Here’s How.

Experiencing so called ms, a situation where one’s immune system is attacking and damaging the nervous system, is something many wish to prevent. Fortunately ms is completely preventable. Here are some of the basics of doing that.

Understanding how to prevent ms involves understanding why it happens. To a large degree, ms is a trigger and response problem. Something triggers the immune system and the immune system responds by attacking the nervous system. So ms prevention involves working the problem from both sides, by reducing triggers and changing the response.

First let’s look at the trigger side.

While, there are numerous, even infinite, different things that can trigger an autoimmune response, fortunately one can generally figure out what many of them might be by using a little common sense.

For instance, substances that tend to cause damage to the body can be a trigger for an autoimmune response. These can include pesticides, certain artificial food ingredients, msg and other chemicals. In one case, a person who was actually making a point of eating in a healthy way found that leaking breast implants seemed to have been triggering auto immune problems. In another, heavy use of tooth care products seemed linked. Cleaning products

and things like dryer sheets can contain immune system triggering chemicals. Even certain medications could potentially trigger an immune system response.

Another thing that can trigger an autoimmune response is a virus or microbe.

What all of these types of triggers have in common is that the fact that they are potentially harmful is no mystery. So preventing ms involves being careful to actually reduce exposure to these triggers by, for instance, eating organically grown high quality food, refraining from using or minimizing use of chemicals on one’s lawn, using less toxic cleaning products, refraining from using dryer sheets and basically living a more natural, simpler lifestyle that does not involve constantly bombarding one’s system with reasons to freak out.

Physical triggers are not the only type though. The immune system can also be triggered emotionally and by being stressed. Once again by using a little common sense one can figure out that if one is living in a stressful way or is constantly emotionally upset, this could contribute to the immune system being unstable and ready to attack.

The answer to this is to do things like work to manage one’s life as well as possible, to reduce stress by resolving issues with others, to learn to stay calm

=======================

**Excerpt 02: "Programmer"**

**Does multiple sclerosis decrease intelligence/IQ?**

Experimental Excerpt:

I'm positive my IQ has dropped drastically. It used to be 157. My memory was like a steel trap. I was at the top of my game as a programmer. Considered one of the best. Now, they triple check everything I do. I use to have 10 projects going on at the same time. Now they barely give me 2 and I am watched over all the time.  So, I think the answer is YES. Next to the pain, I think this is my most devastating symptom. (to me)

89 words, 117 syllables, 336 characters w/o spaces reading time: 27 seconds

Original:

https://www.dailystrength.org/group/multiple-sclerosis-ms/discussion/so-does-ms-affect-your-iq

I'm positive my IQ has dropped drastically. It use to be 157. My memory was like a steel trap. I was at the top of my game as a programmer. Considered one of the best. Now, they triple check everything I do. I use to have 10 projects going on at the same time. Now they barely give me 2 and I am watched over all the time.

So, I think the answer is YES. Next to the pain, I think this is my most devastating symptoms. (to me)

==================================================

**Excerpt 03: "EBV"**

**Is multiple sclerosis preventable?**

Experimental Excerpt:

The question of MS being preventable has a vague answer.  Multiple sclerosis is said to be hereditary.  Some researchers have linked the Epstein-Barr virus or EBV to multiple sclerosis. Avoiding contact and exposure to this virus might be impossible since the EBV is pervasive. Simple things such proper hand washing and avoiding contact with sick people especially during the cold and flu season may be done to help prevent the acquiring of new EBV.  If you eat more foods that are high in vitamin D, you are more protected against multiple sclerosis.

92 words, 151 syllables, 458 characters w/o spaces reading time: 28 seconds

Original:

https://www.medicaldaily.com/preventing-multiple-sclerosis-it-possible-235071

With so many cases of multiple sclerosis, people have constantly searched for its preventive measures. The question of it being preventable, however, is still left with a vague answer. Experts studying multiple sclerosis cases said that preventing its development may or may not be possible. They cannot come up yet with a definite answer. Why? There have been studies about multiple sclerosis but the information gathered is not yet enough. Experts have to understand this illness first before coming up with an answer on whether or not it is preventable.

Multiple sclerosis is said to be hereditary. Dr. Tanuja Chitnis, MD, an assistant professor of neurology and also director of the Partners Pediatric MS Center at Massachusetts General Hospital for Children, was noted saying that 25 per cent of people with identical twins who have multiple sclerosis develop this illness as well. Dr. Chitnis the occurrence of multiple sclerosis in general population is 1 in every 100,000. On the other hand, 5 out of 100 people with siblings, parents or children who suffer from multiple sclerosis end up suffering from this. With a few more studies, experts will soon find out what these families can do to decrease the chances of acquiring the condition.

Some researchers have linked the Epstein-Barr virus or EBV to multiple sclerosis. Epstein-Barr virus is a herpes-related virus. Researchers, however, cannot conclude this theory. The EBV is common to most people. In United States, more than 95 per cent people aged 35 to 40 have acquired this virus at some point. Avoiding contact and exposure to this virus might be impossible since the EBV is pervasive. Simple things such proper hand washing and avoiding contact with sick people especially during the cold and flu season may be done to help prevent the acquiring of EBV. People should be more hygienic by washing their hands regularly especially when you rode on public vehicles.

More studies show that the acquisition of multiple sclerosis depends on the person’s demographic location. The Partners Multiple Sclerosis Center said that there are more incidents and cases of multiple sclerosis in southern Australia, North America and northern Europe. This finding suggests that the farther you are from the equator, the greater your chances are of acquiring multiple sclerosis.

It doesn’t necessarily mean that you should move to another place to avoid developing multiple sclerosis. It is assumed that the lack of vitamin D contributed to the development of multiple sclerosis to people in North America, southern Australia and northern Europe. An increase of vitamin D intake may help since vitamin D was found out to play an important role in protecting against multiple sclerosis. This will boost your immune system and help your body absorb more calcium. Eating healthy is also vital in avoiding multiple sclerosis. If you have to eat more foods which are high in vitamin D, you are more protected against multiple sclerosis according to Dr. Chitnis. Drinking green tea is also said to help lower the risk of having multiple sclerosis.

=====================================

**Excerpt 04: "Avonex"**

**How risky is Tecfidera as a treatment for Multiple Sclerosis?**

Experimental Excerpt:

I was an Avonex patient for about 16 years. Getting tired of injecting Avonex, I asked my doctor to switch me to tecfidera tablets. Within six months my immune system bottomed out. I had to go off the drug and back on an injectable. It took a good six months or more for my immune system to recover. My neurologist never lost sight or control; he was monitoring my blood levels the whole time.

74 words, 108 syllables, 320 characters w/o spaces reading time: 22 seconds

Original:

https://www.quora.com/How-risky-is-Tecfidera-as-a-treatment-for-MS

I was an Avonex patient for about 16 years. Getting tired of injections I asked my doctor to switch me to tecfidera. Within six months my immune system bottomed out. I had to go off the drug and back on an injectable. It took a good six months or more for my immune system to recover. My neurologist never lost sight or control he was monitoring my blood levels the whole time.

========================================

**Excerpt 05: "Up there"**

**How risky is Tecfidera as a treatment for Multiple Sclerosis?**

Experimental Excerpt:

Tecfidera is "up there" in the list of MS medicines that carry some moderate to severe risk, as Tecfidera is one of the MS medicines which can cause PML (progressive multifocal leukoencephalopathy).  PML is usually fatal, but survival rates are improving.  One estimate says that the mortality rate is around 30-50% in the first few months after diagnosis. If you do decide to start Tecfidera, make sure you get annual JC virus tests.  Whether or not you have been exposed to the JC virus should factor in to your decision as to which medicine to take.

96 words, 158 syllables, 454 characters w/o spaces reading time: 30 seconds

Original:

https://www.quora.com/How-risky-is-Tecfidera-as-a-treatment-for-MS

Tecfidera is "up there" in the list of MS medicines that carry some moderate to severe risk, as Tecfidera is one of the MS medicines (along with Tysabri and, quite possibly, Gilenya, although that is still being determined) which can cause PML ([progressive multifocal leukoencephalopathy](https://en.wikipedia.org/wiki/Progressive_multifocal_leukoencephalopathy)). PML is a fast-moving viral infection caused by the [JC virus](https://en.wikipedia.org/wiki/JC_virus) and exacerbated by certain medications and treatment regimems for certain diseases such as AIDS and MS. PML is usually fatal, but survival rates are improving. [One esitmate](http://www.ninds.nih.gov/disorders/pml/pml.htm) says that the mortality rate is around 30-50% in the first few months of identification of the disease.

Tecfidera can also cause allergic reactions and GI symptoms.

Talk to your neurologist about all your treatment options and your entire treatment regimen. If you do decide to start Tecfidera, make sure you get (at least) annual JC virus tests (unless you're positive for JC, then you only need to have it done once). Whether or not you have been exposed to the JC virus should factor in to your decision as to which medicine to take.

===================================================

**Excerpt 06: "Exercises"**

**Does multiple sclerosis decrease intelligence/IQ?**

Experimental Excerpt:

Do not despair. Many people with MS experience cognitive symptoms like short term memory, ability to quickly process information and so on. Something to think about is doing brain exercises. Every day. There are plenty of websites for brain exercises out there. These sites usually offer some of the exercises free, but charge if you want to track your progress or use other brain enhancing exercises. The websites offer exercises and/or games in thinking flexibility, information processing, memory, math, logic, and so on. Good Luck.

85 words, 151 syllables, 451 characters w/o spaces reading time: 25 seconds

Original:

https://www.dailystrength.org/group/multiple-sclerosis-ms/discussion/so-does-ms-affect-your-iq

Do not despair. Many people with MS experience cognitive symptoms like short term memory, ability to quickly process information and so on. Something to think about is doing brain exercises. EVERY DAY. I use the website [Lumosity.com](http://lumosity.com/) and [FitBrains.com](http://fitbrains.com/), but there are plenty out there. Some are available for the smart phone. These sites usually offer some of the exercises free, but charge if you want o track your progress or use other brain enhancing exercises. The websites offer exercises and/or games in thinking flexibility, information processing, memory, math, logic, and so on. The Lumosity site offers a vision field test. Good Luck."

**=================================================**

**Excerpt 07: "Vit D"**

**Is multiple sclerosis preventable?**

Experimental Excerpt:

We know that Vitamin D has a prophylactic effect against MS. We know that in countries north of 40 degrees latitude, there is a much higher prevalence of MS, and researchers believe this may be caused by a decrease in exposure to direct sunlight, which is a major cause for vitamin D deficiency.  Studies have also shown us that the vast majority of people who develop MS are deficient in Vitamin D. We know that vitamin D has a prophylactic effect, but we do not know to what extent, how it works, or what level of vitamin D is enough.

100 words, 152 syllables, 436 characters w/o spaces reading time: 30 seconds

Original:

https://www.quora.com/Is-multiple-sclerosis-preventable

We know that Vitamin D has a prophylactic effect against MS. We don't have to know the cause of MS to know that it works, we have proven this through *epidemiological* studies, which is the study of disease patterns.

We know that above 40 degrees latitude there is a much higher prevalence of MS, and researchers believe this may be caused by a decrease in exposure to direct sunlight, which is a major cause for vitamin D deficiency. Studies have also shown us that the vast majority of people who develop MS are deficient in Vitamin D.

We know that vitamin D has a prophylactic effect, but we do not know to what extent, how it works, or what level of vitamin D is enough.

We also do not know at what age you have to start fixing your vitamin D levels. We do know through epidemiological studies that when you move to a new region, you take on the risk factors of that new region. If you move before age 15, the effects of the new region are seen in the current generation. If you move after age 15, the effects are seen in the next generation. So there is some evidence that if you are not exposed to the positive benefits of a new region before age 15, you retain the same risk factors with which you grew up.

This may mean that a person who was vitamin D deficient their entire life (up to age 15) may not benefit from correcting vitamin D levels now. Again, there is much we do not know.

**====================================================**

**Excerpt 08: "Small Risk"**

**How risky is Tecfidera as a treatment for Multiple Sclerosis?**

Experimental Excerpt:

There may be a small risk of developing progressive multifocal leukoencephalopathy (PML), which is a rare viral disease of the brain. Three cases of PML have been reported in people taking Tecfidera (dimethyl fumarate). The risk of developing PML on Tecfidera is considered very low but if the immune system is weakened and the body is less able to fight an infection, the virus can reactivate.   A blood test can detect the presence and level of the viral antibodies.  If you are worried, discuss your concerns with your MS team.

90 words, 146 syllables, 438 characters w/o spaces reading time: 27 seconds

Original:

https://my-ms.org/med_pml_tysabri.htm

PML and DMTs

We are including this section due to the possible risk of contracting progressive multifocal leukoencephalopathy (PML) while taking Tysabri and other disease-modifying therapies (DMTs) that are used to specifically treat multiple sclerosis (MS).

Now let this be clearly understood. The mentioning of the following medications should in no way make your treatment choice for you, good or bad. Every medication has side effects of one kind or another, severity from mild to extreme, and some just don't work on every person. The medications that we are discussing below are extremely effective in the treatment of MS, and can work perfectly fine for many people. All we are doing here is making sure that you are aware of a possibly severe side effect, that being PML. And the best way to make a fully informed decision on a proper treatment for you, and nobody else, is to learn as much about each medication and discuss each option with your doctor.

Tysabri and PML

Tysabri increases the risk of PML, an opportunistic viral infection of the brain that usually leads to death or severe disability. Risk factors for the development of PML include duration of therapy, prior use of immunosuppressants, and presence of anti-JC Virus antibodies. These factors should be considered in the context of expected benefit when initiating and continuing treatment with Tysabri.

Because of this risk of contracting PML while taking Tysabri, we are providing this you with this information. This isn't meant to make anyone's decision on which treatment to begin or end, but rather to provide any pro's or con's so whatever the decision is, it can be an informed decision. If the risk was a headache, rash, or even gas, that wouldn't be a concern. PML usually happens in individuals with weakened immune systems, however, no one can predict who will get PML. The chances may be higher if people are also being treated with medications that weaken their immune system, including other MS treatments.

Tysabri also increases the risk of developing encephalitis and meningitis caused by herpes simplex and varicella zoster viruses. Serious, life-threatening, and sometimes fatal cases have been reported in the postmarketing setting in MS patients receiving Tysabri. Other serious adverse events that have occurred in Tysabri-treated patients include hypersensitivity reactions (e.g., anaphylaxis) and infections, including opportunistic and other atypical infections. Clinically significant liver injury has also been reported in patients treated with Tysabri in the post-marketing setting. A list of adverse events can be found in the full Tysabri product labeling for each country where it is approved.

If Tysabri is possibly a cause or contributor to those few who have contracted PML, then it must be mentioned. It needs to be understood that no treatment, medication, or even life in general is 100% safe, and it's important to know what one's risks are prior to making any decision. This is not saying that Tysabri should not be considered, but rather that it must be considered carefully as a treatment option and discussed with one's doctor and family. The use of any medication has a risk along with it and that specific risk is never the same in any two people.

Because of the increased risk of PML, it's generally recommended only for patients who have had an inadequate response to, or are unable to tolerate alternate MS therapies. Before you receive your first dose of Tysabri, your doctor should have an MRI taken to make sure you don't have any signs of a brain infection. During your treatment, it's extremely important that your doctor check you every 3 to 6 months to make sure you are not developing any signs of serious infection.

If Tysabri is used alone to treat MS, there is still a chance of getting PML. The chance of getting PML increases with a longer period of treatment. Tysabri may increase your chance of getting an unusual or serious infection, because it can affect your immune system. This can make it easier for you to bleed from an injury or get sick from being around others who are ill. Avoid contact with people who have colds, the flu, or other contagious illnesses and contact your doctor immediately if you develop signs of infection.

Some doctors have also started giving patients a holiday (short period without any treatment) from Tysabri in an effort to avoid PML. In such scenarios, a patient takes Tysabri for two years before taking a break from the drug and turning to other therapies if needed for treatment.

Just know that whatever medication any patient chooses, there is always a chance that it may or may not work for them. There is also a chance of side effects associated with that medication that will more than likely differ with every person taking it. It comes down to how risk adverse each person is and the outcome they expect from each treatment.

Healthcare professionals should monitor patients on Tysabri for any new sign or symptom that may be suggestive of PML. Tysabri dosing should be withheld immediately at the first sign or symptom suggestive of PML. For diagnosis, an evaluation including a gadolinium-enhanced MRI scan of the brain and, when indicated, cerebrospinal fluid analysis for JC viral DNA are recommended.

Gilenya and PML

Three cases of PML have been reported in people taking Gilenya (fingolimod). The risk of developing PML on Gilenya is considered very low but if you are worried, discuss your concerns with your doctor.

Before you start taking Gilenya, it's important that you have had a recent MRI scan. When you start treatment, you should be informed of the early signs and symptoms of PML. These can be similar to an MS relapse, so it's important to report any new or worsening symptoms. If PML is suspected during treatment, an MRI scan should be performed and Gilenya suspended until PML has been excluded.

Tecfidera and PML

Three cases of PML have been reported in people taking Tecfidera (dimethyl fumarate). The risk of developing PML on Tecfidera is considered very low but if you are worried, discuss your concerns again with your doctor.

Before you start taking Tecfidera, it is important that you have had a recent MRI scan. When you start treatment, you should be informed of the early signs and symptoms of PML. These can be similar to an MS relapse, so it is important to report any new or worsening symptoms. If PML is suspected during treatment, an MRI scan should be performed and Tecfidera suspended until PML has been excluded. The cases of PML have occurred in people who have had very low levels of lymphocytes (a type of white blood cell) for an extended period of time. Once you've started treatment you should have blood tests every three months to monitor your blood cell counts.

Other Medications and PML

Tysabri, Gilenya and Tecfidera are not the only drugs that has connections to PML. There have been several other drugs that have been linked to the deadly PML brain infections in recent years. The psoriasis drug Raptiva was recalled in April of 2009 due to PML infections, and the cancer drug Rituxan was recently connected to 57 cases of PML infection in a study published in the May 2009 issue of the medical journal Blood.

====================================================

**Excerpt 09: "Half"**

**Does multiple sclerosis decrease intelligence/IQ?**

Experimental Excerpt:

Cognitive changes are common in people with MS — approximately half of all people with MS will develop problems with cognition. Cognition refers to a range of high-level brain functions, including the ability to learn and remember information; organize, plan and problem-solve; focus, maintain and shift attention as necessary; understand and use language; accurately perceive the environment; and perform calculations. In MS, certain functions are more likely to be affected than others.

71 words, 134 syllables, 417 characters w/o spaces reading time: 21 seconds

Original

http://www.ms.pitt.edu/symptoms/cognitive-change

Cognitive changes are common in people with MS — approximately half of all people with MS will develop problems with cognition. Cognition refers to a range of high-level brain functions, including the ability to learn and remember information; organize, plan and problem-solve; focus, maintain and shift attention as necessary; understand and use language; accurately perceive the environment; and perform calculations. In MS, certain functions are more likely to be affected than others:

- Memory (acquiring, retaining and retrieving new information)
- Attention and concentration (particularly divided attention)
- Information processing (dealing with information gathered by the five senses)
- Executive functions (planning and prioritizing)
- Visuospatial functions (visual perception and constructional abilities)
- Verbal fluency (word-finding)

=========================================
