# Supplementary material for: Spelling Errors and Shouting Capitalization Lead to Additive Penalties to Trustworthiness of Online Health Information: Randomized Experiment With Laypersons
Source: J Med Internet Res. 2020 Jun 10;22(6):e15171. doi: 10.2196/15171 (PMC7315370; doi:10.2196/15171)
Supplement: Multimedia Appendix 5 [file jmir_v22i6e15171_app5.docx]

Checklist reporting of survey cherries for incivility trustworthiness study Witchel

[Who and What This Is 2](#_Toc9429968)

[Reference for CHERRIES 2](#_Toc9429969)

[Describe survey design 2](#_Toc9429970)

[IRB approval 2](#_Toc9429971)

[Informed consent 2](#_Toc9429972)

[Data protection 2](#_Toc9429973)

[Development and testing 2](#_Toc9429974)

[Open survey versus closed survey 3](#_Toc9429975)

[Contact mode 3](#_Toc9429976)

[Advertising the survey 3](#_Toc9429977)

[Web/E-mail 3](#_Toc9429978)

[Context 3](#_Toc9429979)

[Mandatory/voluntary 3](#_Toc9429980)

[Incentives 3](#_Toc9429981)

[Time/Date 3](#_Toc9429982)

[Randomization of items or questionnaires 3](#_Toc9429983)

[Adaptive questioning 3](#_Toc9429984)

[Number of Items 3](#_Toc9429985)

[Number of screens (pages) 3](#_Toc9429986)

[Completeness check 3](#_Toc9429987)

[Review step 3](#_Toc9429988)

[Unique site visitor 3](#_Toc9429989)

[View rate (Ratio of unique survey visitors/unique site visitors) 3](#_Toc9429990)

[Participation rate (Ratio of unique visitors who agreed to participate/unique first survey page visitors) 3](#_Toc9429991)

[Completion rate (Ratio of users who finished the survey/users who agreed to participate) 3](#_Toc9429992)

[Cookies used 3](#_Toc9429993)

[IP check 3](#_Toc9429994)

[Log file analysis 3](#_Toc9429995)

[Registration 3](#_Toc9429996)

[Handling of incomplete questionnaires 3](#_Toc9429997)

[Questionnaires submitted with an atypical timestamp 3](#_Toc9429998)

[Statistical correction 3](#_Toc9429999)

[Advertisement 3](#_Toc9430000)

[A psychology study about the trustworthiness of what you read on the web. 3](#_Toc9430001)

[Requirements 3](#_Toc9430002)

[Keywords 3](#_Toc9430003)

[About the researcher 3](#_Toc9430004)

[Ethical approval 3](#_Toc9430005)

## Who and What This Is

This is the reporting of the questionnaires for the BSMS surveys on trustworthiness of online health information relating to multiple sclerosis by HJ Witchel et al. This is in the CHERRIES format.

## Reference for CHERRIES

Eysenbach G. Improving the quality of Web surveys: the Checklist for Reporting Results of Internet E-Surveys (CHERRIES). Journal of Medical Internet Research. 2004;6(3):e34.

## Describe survey design

This was an open survey with a convenience sample of healthy adult participants. It was advertised on a website for people who want to take part in scientific studies. On the advertising and in the ethics of the study there are statements that non-adults and people from vulnerable populations should not participate.

## IRB approval

This study received institutional approval from Brighton and Sussex Medical School's Research Governance and Ethics Committee (within the University of Sussex). The approval number is 16/044/WIT and that approval letter date is 14 October 2016.

## Informed consent

The informed consent was the first page of the online study. This page was estimated to take 2 minutes to read. On both the informed consent page and the advertisement we stated that "We estimate that this questionnaire will take 8-10 minutes to complete". On the landing page, this was the first statement after the title. The survey told/warned the participants that it was an online survey, that we were not the only holders of the data, and that they should not submit sensitive personal data if they were uncomfortable with this. We did not collect sensitive data (except demographics, see below), although we allowed participants to include their email address if they wanted to receive information about the study later. We told participants that the data would be kept by our team for 5 years, but that we could not predict how long our online company would keep the data. The primary investigator (and his email address) was included as well as the name of Brighton and Sussex Medical School. The instructions included the following goals statement: " GOALS: By performing this survey you will be helping us to understand how people online read and make decisions about trust."

## Data protection

The survey included demographic data: age, gender, profession, and when the participant learned to speak English (i.e. were they a native English speaker). This data is maintained online by Qualtrics, for five years. After downloading, the data was transferred to Excel files with a password that were stored on password protected computers. These computers are all behind locked doors.

## Development and testing

Qualtrics is a well-established survey presentation platform. The initial version of our survey was developed by three investigators, with a small usability test as follows: three friends of one of the investigators who were unaware of the purpose of the study, and they took the survey and were immediately asked for oral feedback according to a structured set of questions about their comprehension and any problems they had.

## Open survey versus closed survey

This was an open survey.

## Contact mode

The initial contact with participants was made online. This was done via advertising that they had to seek out on prolific.co

## Advertising the survey

The advertisement is at the bottom of this. This advertisement appeared on the prolific.co website. Prolific.co is an organisation that draws upon people who want to participate in research.

## Web/E-mail

The survey was on the Qualtrics website. Qualtrics is a provider of survey software, and many psychology questionnaires are hosted by it. The advertisements were on the prolific.co website and its associated advertisements. The result would be that we should receive people who are interested in participating in psychology experiments (or people who wanted the incentive).

## Context

The survey was on the Qualtrics website. Qualtrics is a provider of survey software, and a lot of psychology questionnaires are hosted by it. The advertisements were on the prolific.co website and its associated advertisements. The result would be that we should receive people who are interested in participating in psychology experiments (or people who wanted the incentive).

## Mandatory/voluntary

The survey was voluntary.

## Incentives

we offered incentives to complete the questionnaire: £1.

## Time/Date

The surveys were done in late August 2019.

## Randomization of items or questionnaires

The order of the 9 test paragraphs were randomised, as were the versions/conditions for each of these paragraphs (no errors, misspellings, capitalisations, both misspellings and capitalisations). The conditions were counter-balanced, so that over time we received approximately equal numbers of each.

## Adaptive questioning

Not done.

## Number of Items

Each item was presented on a separate screen (except for demographics). There were four demographic items (approx age, gender, profession, and knowledge of English language), and then 11 paragraphs to read and judge. The first two paragraphs were always the same (training), and were not included in the analysis, although these training paragraphs were not marked as different in any way. We included them to help the participants understand the nature of the test.

## Number of screens (pages)

In addition to the 12 screens of excerpts and demographics, there was the landing page with ethical explanation, and instructions page on what should happen during the task, and an explanation/outro page at the end that allow participants to include their email address if they wanted to receive information later about the project.

## Completeness check

Participants could not move on to the next item without completing the current item (although they could of course abandon the survey at any time). All demographic items included an option for "rather not say". The completeness check was performed by Qualtrics using JAVAScript.

## Review step

There was no way to review their answers. This worked for our purposes because we collected semantic differential data and we wanted participants' instinctive response to the excerpt.

## Unique site visitor

Prolific.co maintains accounts with its participants, and we requested that its participants only interact with any of our surveys only once.

## View rate (Ratio of unique survey visitors/unique site visitors)

We do not know the view rate, which would be the number of participants (from Qualtrics) divided by the number of visitors to the advertising sites (eg prolific.co).

## Participation rate (Ratio of unique visitors who agreed to participate/unique first survey page visitors)

The recruitment rate for the three editions of the survey was 100%.

## Completion rate (Ratio of users who finished the survey/users who agreed to participate)

87%.

## Cookies used

N/A

## IP check

IP was checked. It was maintained for 24 hours.

## Log file analysis

No analysis was performed.

## Registration

N/A

## Handling of incomplete questionnaires

Incomplete questionnaires were not included in the analyses.

## Questionnaires submitted with an atypical timestamp

Time to complete the survey was included in the log file. We removed from the analysis any participants who spent less than 80 seconds on the survey, or who took longer than 20 minutes.

## Statistical correction

N/A

## Advertisement

Advert on prolific.co 2019-08-20

A psychology study about the trustworthiness of what you read on the web.

How trustworthy do you find what you read on the web?

Please take part in our online psychology experiment lasting ~8 minutes. The task involves rating short paragraphs about how trustworthy you find them. This is approved university research; non-commercial, with informed consent. All data is confidential and anonymised.

## Requirements

Over 18

## Keywords

Brighton and Sussex Medical School

Trust

psychology

credibility

text

paragraphs

information

web

online

## About the researcher

Our team of researchers investigate the psychology of credibility and engagement -- particularly when people are interacting with a computer or online device. You can read a summary of one of our previous research projects (as described by Scientific American) here:

http://www.scientificamerican.com/article/now-computers-can-tell-when-you-re-bored/

## Ethical approval

This study has been approved by University of Sussex (UK) Research Governance and Ethics Committee (RGEC) on 6 February 2017.

Brighton and Sussex Medical School, GB

8 min(s) to complete

Prize draw of three £50 Amazon gift certificates

Online Questionnaire

Compensation

Prize draw of three £50 Amazon gift certificates

Study type

Online Questionnaire

How long will the study take to complete?

8 minutes

Study language

English

Participant requirements

Over 18

Online survey

Instructions

Follow the link to take part in this 8 minute online questionnaire that involves rating short paragraphs about how trustworthy you find them.

This survey takes approximately 8 minutes to complete. When you arrive at the website, you will be asked to fill in a small amount of demographic information (approximate age group, etc.). Then you will read a series of short paragraphs that you might read on the web. For each paragraph you will rate how much you feel it is trustworthy or untrustworthy. At the end you can leave your email address if you wish to participate in the prize draw (or receive more information), but this step is not required.

Maximum Number of Participants

320
